# The Leukocyte VCS Parameters Compared with Procalcitonin, Interleukin-6, and Soluble Hemoglobin Scavenger Receptor sCD163 for Prediction of Sepsis in Patients with Cirrhosis

**DOI:** 10.1155/2019/1369798

**Published:** 2019-12-12

**Authors:** Fan Guo, Yang-chun Feng, Gang Zhao, Hui-li Wu, Ling Xu, Jing Zhao, Jie Lv, Song-tao Han, Yan-chun Huang, Xiu-min Ma

**Affiliations:** ^1^State Key Laboratory of Pathogenesis, Prevention and Treatment of High Incidence Diseases in Central Asia, The First Affiliated Hospital of Xinjiang Medical University, Urumqi 830000, China; ^2^Clinical Laboratory Center, Tumor Hospital Affiliated to Xinjiang Medical University, Urumqi 830000, China; ^3^Department of Blood Transfusion, Affiliated Traditional Chinese Medicine Hospital of Xinjiang Medical University, Urumqi 830000, China; ^4^Intensive Care Unit, Affiliated Traditional Chinese Medicine Hospital of Xinjiang Medical University, Urumqi 830000, China; ^5^Clinical Laboratory Center, The First People's Hospital of Huaihua, Huaihua 418000, China

## Abstract

**Background:**

Patients with liver cirrhosis have a high risk of sepsis and a poor prognosis. Recently, a new standard for sepsis (Sepsis-3) has been proposed in the general population. The Coulter Lh 750 hematology analyzer can evaluate mean volume, conductivity, scatter, and distribution width of leukocyte. We tried to use Sepsis-3 criteria to study the diagnostic value of volume, conductivity, and scattering (VCS) parameters in sepsis and infection in patients with liver cirrhosis compared with traditional infection markers (PCT, IL-6, sCDl63).

**Methods:**

A blinded, cohort study was conducted in three different ED populations within three affiliated hospitals. A total of 249 patients with liver cirrhosis were enrolled in the study. According to the “Sepsis-3” consensus criteria, clinical history, and laboratory examination, the subjects were divided into sepsis (*n* = 54), patients with infections (*n* = 95), and patients without systemic infections (*n* = 100). The blood samples of the patients were collected at the time of ED admission and were evaluated for the detection of sepsis.

**Results:**

The differences of MNV, MNS, MMV, MMS, MLV, NDW, and MDW in the three groups were statistically significant. In the diagnosis of sepsis in patients with liver cirrhosis, the sensitivity of combined detection of MMV and MDW was 88.89%; the specificity was 74%. This sensitivity was significantly better than the 83.3% achieved using 0.97 mg/L as the cutoff for sCD163. In the diagnosis of infection in cirrhosis, the sensitivity of combination of MNV and MMS was increased to 86.32%; the specificity was 92%. The sensitivity was the same as that achieved by using 0.31 ng/mL as the cutoff value of PCT, but the specificity increased.

**Conclusion:**

The leukocyte VCS parameter could be potential parameters for indicating sepsis and infection in patients with liver cirrhosis. The combined detection of MMV and MDW seemed to be helpful for the diagnosis of sepsis in these patients, and the combination of MNV and MMS could better indicate infection for them.

## 1. Introduction

Patients with cirrhosis are susceptible to infection and sepsis because of immunological abnormalities [1]. When sepsis occurs, the mortality rate of cirrhotic patients increases four times [2]. Therefore, the early diagnosis of sepsis in cirrhotic patients is crucial. However, the systemic inflammatory response syndrome (SIRS) criteria for the diagnosis of sepsis in patients with liver cirrhosis and infection have poor accuracy. In fact, patients with liver cirrhosis may have (1) tachypnea induced by encephalopathy, (2) leukopenia caused by hypersplenism, and (3) bradycardia due to the use of beta-blockers [3, 4]. These factors may impair SIRS parameters.

Sepsis is currently defined as organ dysfunction that is life-threatening due to an imbalance in the body's response to infection [5]. For the definition of Sepsis-3, the “Surviving Sepsis Campaign” recommends that the first step in identifying and treating sepsis is to identify “suspected infections” [6]. The early detection and treatment of sepsis remains a major clinical challenge. Infection is usually detected more than 4 hours after admission [7]. Biomarkers play an important role in the early diagnosis of sepsis, the judgement of condition and prognosis, and the evaluation of curative effect, such as the detection of procalcitonin (PCT), interleukin-6 (IL-6), and white blood count (WBC). However, PCT and IL-6 are not usually used for early detection. Unfortunately, leukopenia is nonspecific in patients with cirrhosis, and some even have leukopenia due to hypersplenism. Therefore, there is an urgent need for a conventional biomarker in the assessment of their infections in ED to enhance the detection of sepsis and infection.

The Coulter LH750 hematology analyzer (Beckman Coulter, Fullerton, CA) use the volume, conductivity, and scatter (VCS) technology to analyze WBC in the near original state. In addition to detecting routine blood cell parameters, it can also provide quantitative WBC volume, conductivity, and light scatter to reflect the morphological changes of them. Recent studies have shown that the increase in the volume of rudimentary immune cells reflected in the VCS parameter may help detect sepsis [8–12]. However, the application of VCS parameters in sepsis for patients with cirrhosis was less studied. Therefore, we aimed to elucidate the applicability of VCS parameters as potential predictors for infections in patients with cirrhosis and compare their reliability with traditional inflammatory biomarkers such as PCT, IL-6, and soluble hemoglobin clearance receptor (sCD163).

## 2. Materials and Methods

### 2.1. Patient Samples

This is a retrospective analysis of prospective data collected in a cohort study, which included consecutive subjects admitted to the EDs of three affiliated hospitals of Xinjiang Medical University from February 2018 to June 2019 due to complications of cirrhosis. The inclusion criteria were diagnosis of liver cirrhosis according to histology, clinical, biochemical, imaging, or endoscopic findings. Patients were excluded from (1) selective hospitalization, (2) admissions unrelated to complications of liver cirrhosis, (3) criteria for hepatocellular carcinoma (HCC) outside Milan, (4) extrahepatic malignant tumors, (5) severe extrahepatic diseases, (6) HIV positive patients, and (7) use of immunosuppressive drugs. All patients were initially sent to the EDs. Depending on the severity of the complications, the attending physician decides to transfer the patient to the ward or intensive care unit (ICU).

A total of 257 patients with cirrhosis were originally enrolled into the study. At the time of admission to ICU, the disease was diagnosed by three attending clinicians. Fifty-eight (*n* = 58) patients with cirrhosis fulfilled the criteria of sepsis according to the “Sepsis-3” consensus criteria [5]. Sepsis criteria had clear or suspected basis for infection and met the following indicators: temperature > 38.3°C or <36°C, WBC > 12000/mm^3^ or< 4000/mm^3^ or the proportion of immature cells exceeds 10%, heart rate > 90 beats/min, and blood lactate > 1 mmol/L; two of the above criteria were met, and the bedside rapid sequential organ failure score (qSOFA score) needs to meet at least two of the following: respiratory rate > 22 times/min, Glasgow Coma score (GCS) score < 15, and systolic blood pressure ≤ 100 mmHg. Ninety-seven (*n* = 97) patients suffered from limited organ-specific infections, such as abdominal, pulmonary, gastrointestinal, and urinary tract infections. Pulmonary infection was diagnosed by clinical symptoms (temperature > 38.5°C; purulent sputum), laboratory examination and positive chest X-ray examination; abdominal infection by bacterial/fungal infection evidence and clinical symptoms diagnosis; gastrointestinal infection by clinical symptoms and other laboratory tests of vomit, feces to diagnose; and infection of urinary system by clinical signs and positive urine culture of important pathogens. The controls were randomly selected from patients with cirrhosis without signs of sepsis and infection. All patients had blood culture tests. At the same time, data of WBC counts and related parameters were collected, and blood specimen was collected for the detection of other inflammatory markers. The presence of infection was determined by using all available clinical data in 7 days after ED admission, including results from cultures, blood, and related imaging. Sepsis criteria must be completed within 6 hours of blood cell testing, and the above groups are reviewed by superior experts through electronic medical records.

If the data collection was incomplete (e.g., due to blood collection failure, because blood cultures yielded bacteria likely to be contaminants), the patient was excluded from the statistical analysis. Therefore, the actual number of subjects who underwent data analysis was *n* = 54 for septic patients (group 1), *n* = 95 for infected patients (group 2), and *n* = 100 for patients with nonsepsis and no infections (group 3). This study was in accordance with the ethical guidelines of the 1975 Declaration of Helsinki and approved by the ethics committee of Affiliated Traditional Chinese Medicine Hospital of Xinjiang Medical University. Written informed consent was obtained from all subjects or their legal representatives before the study.

### 2.2. Blood Culture and Microbial Culture

The culture of secretions was carried out in HF Safe 760S biosafety cabinet (Hong Kong, China). The secretion of the subjects was inoculated with automatic plate inoculation instrument (French Biomerieux) and VITEK Mass Spectrometer (VITEK MS, French Biomerieux). The bacterial identification was carried out by automatic bacteriological identification instrument (VITEK-2 Compact), and the patient's blood culture was carried out by automatic blood culture instrument (French Biomerieux). The positive specimens were then cultured and identified.

### 2.3. White Cell Volume Determination

Data included total WBC count and VCS parameters of neutrophils, monocytes, and lymphocytes were obtained within 2 h after sample collection. The samples were analyzed on Coulter LH 750 (Beckman Coulter, Inc.). Blood detection method was carried out in accordance with the instrument operating procedures. The VCS parameters were generated by each individual cell passing through the aperture, which were optically and electronically measured by the instrument. The parameters of each sample are (1) the mean channel of neutrophil cell volume [given by mean neutrophil volume (MNV)], monocytes (given by MMV), and lymphocytes (given by MLV); (2) conductivity (given by MNC, MMC, and MLC); (3) light scatter (given by MNS, MMS, and MLC); and (4) neutrophil and monocyte volume distribution width (NDW, MDW). During the use of the Coulter LH750, calibration (including VCS calibration), quality control, and maintenance were performed in accordance with the instructions. The attending physician was blinded to these results during the assignment of patients to clinical categories, and the reports they received were complete blood counts and classification of white blood cell. At the same time, the laboratory technicians were blinded to the clinical data.

### 2.4. Procalcitonin and Interleukin-6

Serum procalcitonin and interleukin-6 were determined by using a fully automated electrochemiluminescence immunoassay (cobas e 601; Roche Diagnostica GmbH, Mannheim, Germany). The lowest detectable level of procalcitonin (sensitivity) was estimated to be 0.02 ng/mL, intra-assay precision (CV %) was 3.20, and total accuracy 6.24. For a two-point calibration, the test of procalcitonin was linear within a concentration range of 0.02-100 ng/mL. And detection limit for IL-6 was 1.5-5000 pg/mL, intra-assay precision (CV %) was 4.30, and total accuracy 6.65.

### 2.5. sCD163 Laboratory Tests

The venous blood of the subjects was collected in a yellow BD tube containing coagulant and gel and then centrifuged at 3000 r/min for 15 min within 2 hours after standing at room temperature. The upper serum was transferred to a 1.5 mL sterile Eppendorf tube. The tubes were marked and stored in -80°C refrigerator until testing. Serum sCDl63 was tested in November 2018 and June 2019. The kit for detecting sCDl63 is from the Netherlands IQ company (Macro 163TM soluble ELISA assay, IQP, Groningen, Netherlands), and the concentration of serum sCDl63 is detected by enzyme-linked immunosorbent assay (ELISA). The detection range of the kit is 0.006-31.7 mg/L. Sufficient evidence has confirmed that sCD163 was a highly stable marker that can be stored at -80°C for at least 16 months; it is resistant to repeated freeze-thaw cycles.

### 2.6. Statistical Analysis

Statistical analyses were performed using the SPSS 20.0 statistical software. Results were expressed as the mean ± standard deviation. For comparison of more than two groups, ANOVA was used for normal distributions and the Kruskal-Wallis test for nonnormal distributions. ANOVA and post hoc analyses were used to compare the mean values of each group, and multiple comparisons were carried out in pairs to determine which averages were different. *P* value of <0.05 was deemed to indicate statistical significance. Receiver operating characteristic curve analysis was used to determine the power of variables between the sepsis group and infection group. The area under the curve was calculated and the best cutoff value was obtained by Youden index. Logistic regression analysis was used to compare independent variables and then sensitivity and specificity were calculated for independent variables determined by logistic regression analysis.

## 3. Results

### 3.1. Microbial Culture

More than half of sepsis patients (52%) had positive blood culture results. The results were given in Table 1. Among them, the first three major pathogens were Klebsiella pneumoniae (*n* = 7), Pseudomonas aeruginosa (*n* = 5), and Acinetobacter baumannii (*n* = 4).

85 cases of body fluid and secretion culture were positive in the infection group. The infection sites incorporated abdominal, pulmonary, gastrointestinal, and urinary tract. Among them, 59 strains of Gram-negative bacteria, 11 strains of Gram-positive bacteria, and 15 strains of fungi were isolated and identified. The results of separation and identification are shown in Table 2. The Gram-negative bacteria were mostly Klebsiella pneumoniae and Pseudomonas aeruginosa, accounting for 10.59% and 9.41%. The main fungi were Candida albicans and Candida krsurolimus, which accounted for 4.71% and 3.53% of all isolated bacteria, respectively, and the most Gram-positive bacteria were Staphylococcus aureus (4.71%).

### 3.2. General Demographic Characteristics

Data on age and gender distribution of the study participants are shown in Table 3. In sepsis, the age range was 39-78 years (mean 66.6 ± 16.0 years). In the infection group, the age range was 42-73 years; the average age was 65.5 ± 15.8 years. In the control group, the age range was 40-76 years; the average age was 64.2 ± 16.3 years. In these three groups, the proportion of men was higher than that of women, but there was no statistical difference among these groups (*P* > 0.05). The most common aetiologies of cirrhosis in this cohort were alcohol consumption and HCV infection.

### 3.3. Traditional Infection Markers

**Figure 1:**
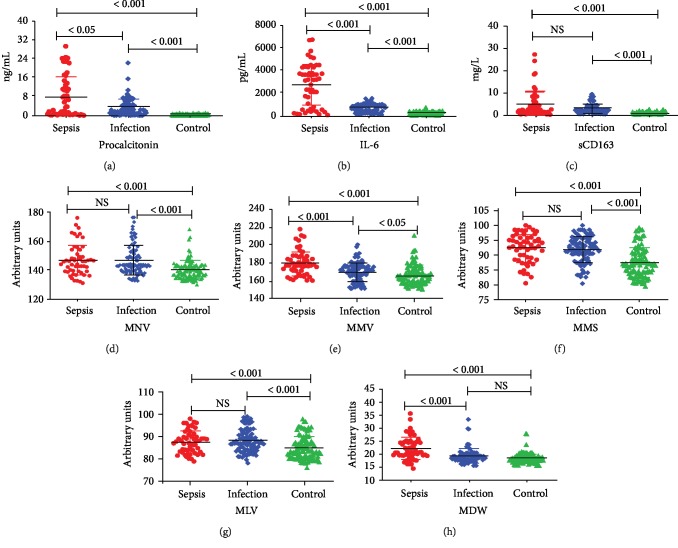
The detection value (mean and SD) of procalcitonin (a), interleukin-6 (b), soluble hemoglobin scavenger receptor sCD163 (c), MNV (d), MMV (e), MMS (f), MLV (g), and MDW (h) in patients with sepsis, infection, and control (significances between groups were calculated with ANOVA and post hoc analyses).

There were significant differences in PCT, IL-6, and sCD163 between three groups (all *P* < 0.001). The levels of PCT, IL-6, and sCD163 in the sepsis group were significantly higher than other two groups (Table 3). In all groups, the concentrations of PCT and IL-6 in the sepsis group were higher than those of the noninfection (*P* < 0.001) and infection groups (*P* < 0.05; *P* < 0.001). Significantly increased concentrations of PCT, IL-6, and sCD163 were observed in the limited infections compared with controls (Figures 1(a), 1(b), 1(c)). A tendency towards higher sCD163 was observed from group 3 to group 1. However, compared with the infection group, it did not reach a significant level (Figure 1(c)).

### 3.4. VCS Parameters

The WBC ranged from 1.4 to 18.3 in sepsis, and the leukocyte count ranges from 1.6 to 19.2 in patients with infection. There was no difference in WBC between the two groups. We evaluated the VCS parameters of all the subjects, and the differences of MNV, MNS, MMV, MMS, MLV, NDW, and MDW in the three groups were statistically significant. MNV was significantly increased in the sepsis and infection groups (Figure 1(d)), while MNS was decreased (Figure 1(d)). For monocytes, MMV was the highest in sepsis compared with the second and third groups (Figure 1(e)). But no difference in average monocyte conductivity (MMC) was observed among them (Table 3). Although there was an increase in monocyte light scatter (MMS) in patients compared with the control groups, no difference in it was observed between the sepsis and infection groups (Figure 1(f)). In lymphocytes, MLV was statistically significant among these groups. Compared with control, the MLV value of the first and second groups increased but there was no significant difference between them (Figure 1(g)). In the cell volume distribution width, NDW and MDW in the septic group were significantly increased (Table 3). The MDW values of patients with cirrhosis in the noninfected group were lower than those of the infected group (Figure 1(h)).

### 3.5. Sensitivity and Specificity of Predicting Sepsis in Patients with Cirrhosis

**Figure 2:**
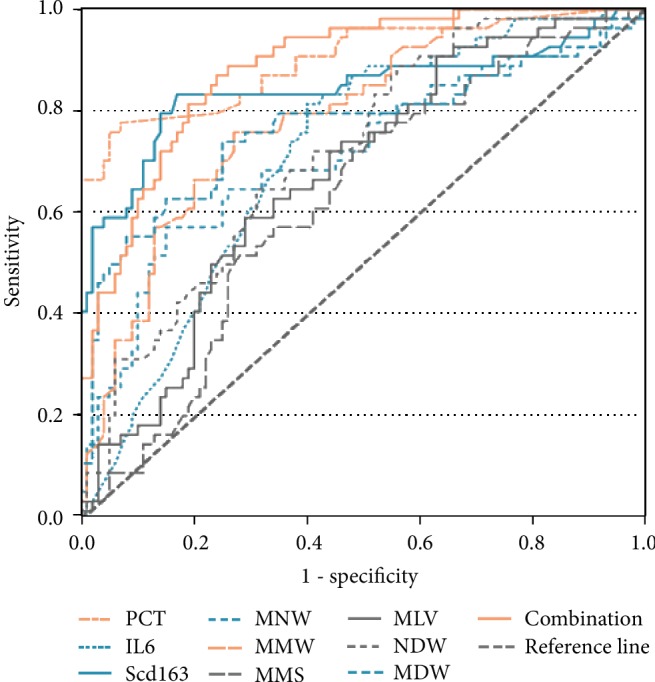
ROC curves (with AUCs) comparing inflammatory markers [procalcitonin (PCT), interleukin-6 (IL-6), soluble hemoglobin scavenger receptor (sCD163), mean monocyte volume (MMV), mean neutrophil volume (MNV), mean monocyte light scatter (MMS), mean lymphocyte volume (MLV), neutrophil volume distribution width (NDW), monocyte volume distribution width (MDW), MMV+MDW (combination)] discriminating between patients with sepsis and control.

Table 4 lists the AUC, sensitivity, specificity, PPV and NPV, and cutoffs for PCT, IL-6, sCD163, MNV, MMV, MMS, MLV, NDW, MDW, and the combination of MMV and MDW. The sensitivity, specificity, PPV, and NPV of the studied parameters were calculated at a specified cutoff value, the cutoff value being taken from the ROC curve (Figure 2). When we selected a cutoff point for MMV greater than 170.33, a sensitivity of 75.9% and a specificity of 73% were achieved. When a cutoff point of MDW greater than 19.19 was selected, the sensitivity and specificity of diagnosis of sepsis were 75.9% and 76%, respectively. With the combination of MMV and MDW, we applied binary logistic regression analysis and established the following regression equation to calculate the probability value PRE (P): Ln (P/1-P): Ln (P/1‐P) = −26.909 + 0.103∗MMV + 0.433∗MDW. The area under the curve of combined detection of MMV and MDW was 0.886, and the sensitivity increased to 88.89%, which was significantly better than the 83.3% achieved using 0.97 mg/L as the cutoff for sCD163.

### 3.6. Sensitivity and Specificity of Predicting Infection in Patients with Cirrhosis

**Figure 3:**
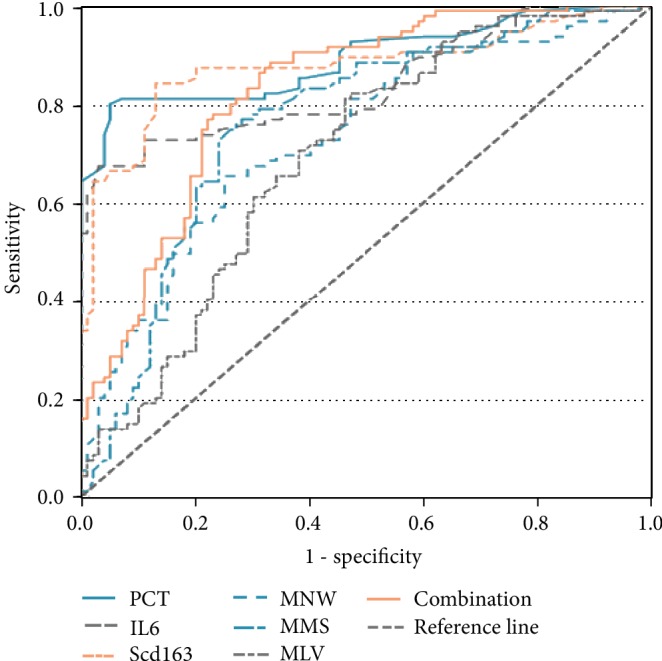
ROC curves (with AUCs) comparing inflammatory markers [procalcitonin (PCT), interleukin-6 (IL-6), soluble hemoglobin scavenger receptor (sCD163), mean neutrophil volume (MNV), mean monocyte light scatter (MMS), mean lymphocyte volume (MLV), MNV+MMS (combination)] discriminating between patients with cirrhosis with infection and control.

The optimal cutoff levels of PCT, IL-6, sCD163, MNV, MMS, and MLV were calculated between the infection group and the noninfected control group by drawing the receiver operating characteristic curves (Figure 3).  Table 5 lists their sensitivity, specificity, PPV, NPV, AUC, and cutoff values. The best cutoff levels of PCT, IL-6, and sCD163 were 0.31 ng/mL, 392 pg/mL, and 0.61 mg/L, respectively. Compared with control, the cutoff levels of MNV, MMS, and MLV in the infected group were >137.81 au, >89.53 au, and >80.875 au, respectively. MNV and MMS were found to be independent risk factors of limited organ-specific infection in logistic regression analysis. With the combination of MNV and MMS, we applied binary logistic regression analysis and established the following regression equation to calculate the probability value PRE (P): Ln (P/1‐P) = −35.969 + 0.116∗MNV + 0.215∗MMS. We found that the area under the curve of the combined detection of MNV and MMS was 0.826, the sensitivity increased to 86.32%, and the specificity was 92%.The sensitivity was the same as that achieved by using 0.31 ng/mL as the PCT's cutoff level, but the specificity was increased.

## 4. Discussion

White blood cells include granulocytes, lymphocytes, and monocyte-macrophages. Neutrophils and monocyte-macrophages in circulating blood have phagocytic effects on bacteria and other foreign bodies. The determination of phagocytic function can be used as an indicator of immune function of the body and can also be helpful for the differentiation of some diseases. The main functions of neutrophils include chemotaxis, adhesion, phagocytosis, and sterilization. Chemotactic function means that it produces directional movement under the action of chemokines and focuses on the loss of inflammation. Adhesion function refers to its adhesion to the natural surface such as the injured vascular wall, receiving information and making corresponding responses to regulate cell behavior. Phagocytosis can be divided into surface phagocytosis and conditioning phagocytosis. Sterilization has two mechanisms: nonoxygen sterilization and oxygen sterilization [13]. Monocytes and phagocytic cells differentiated from monocytes are collectively called monocyte-macrophage systems. Monocyte-macrophages also have tendency, phagocytosis, antitumor activity, regulation of leukocyte formation, and so on. In addition, lymphocytes are also an important cellular component of immune response. Volume increases are an early manifestation that these immune cells respond to severe infections and as such have been shown to serve as potential human biomarkers for latent sepsis [8–12]. Our results show changes in the volume of circulating immune cells, especially the increased mean neutrophil volume (MNV), mean monocyte volume (MMV), mean lymphocyte volume (MLV), and monocyte distribution width (MDW). This is consistent with recent studies on changes in leukocyte VCS parameters in the establishment of patients with cancer undergoing cytotoxic chemotherapy [14].

It has been shown that during acute bacterial infection, reactive and left-shifted neutrophils not only increase in number but also in volume [15], as shown above in the increase in MNV. Therefore, when the body becomes infected, a part of neutrophilic lobular granulocytes and monocyte-macrophages will be chemotaxis, migration, deformation, and phagocytosis, resulting in their inevitably increasing volume. As we have seen, the mean neutrophil volume (MNV) and the mean monocyte volume (MMV) increased for sepsis and local infection. The VCS technology of the Coulter LH750 hematology analyzer can evaluate the biophysical characteristics of more than 8000 white blood cells and calculate the volume distribution width on the basis which can reflect the volume change of all cell types. It uses direct current impedance to measure cell volume (V), radio frequency opacity to characterize its internal conductivity (C), and a laser beam to detect light scatter (S) for nuclear structure and cytoplasmic granularity. These parameters can indicate morphological changes in WBC, which is very important for the study of infection status [16]. VCS parameters have been widely used in the diagnosis of bacterial infection [15], sepsis in emergency patients [17], and leukemia [18] in recent studies. For patients with liver cirrhosis, sepsis and infections increase the risk of organ failures and mortality. At present, there is little research on the variation of VCS parameters in patients with cirrhosis.

Among the traditional biomarkers, complete blood count (CBC), procalcitonin, and IL-6 are most commonly used in the diagnosis of sepsis. Soluble hemoglobin scavenger receptor (sCD163) is a transmembrane protein expressed only on the membrane of mononuclear macrophages [19], which can shed into blood during inflammatory reaction. It has been shown that sCD163 may be an early sensitive marker of sepsis [20]. The detection of the latter three parameters is expensive, and the time of each parameter rising in the body and its half-life time should be taken into account. Laboratory microbial culture is regarded as the “golden standard” for the diagnosis of infection, but it takes a long time (usually 2-3 days) to detect the pathogen. The key difference between VCS parameters and these parameters, as well as other biomarkers of sepsis, is that they are available to ED clinicians at an early stage of clinical evaluation, at a time when the diagnosis of sepsis and infection has not been considered [17].

VCS data derived from the results of blood routine analysis have the advantages of fast, objective, reliable, and so on. They reflect the size of white blood cells, the internal structure of the cells, and the characteristics of granules and nuclear structures in the cytoplasm. In our study, 54 patients with cirrhosis were diagnosed with sepsis, 95 patients had infections, and 100 patients showed no signs of infection. At first, we compared VCS parameters among the three groups. We found that not only the VCS parameters of neutrophils could indicate the presence of infection but also the VCS parameters of monocytes and lymphocytes were significantly different (Table 3). This phenomenon suggested that when sepsis and local infections had occurred, neutrophils, monocytes, and lymphocytes became larger in size, as newborn cells tended to be immature and larger. In addition, MDW increased in the septic and locally infected groups because circulating monocytes were the first responders to infections [21, 22] and the response was proportional to the intensity of exposure to bacteria and fungi [23]. This led to a sharp increase in monocyte size [24], which has not been shown in the monocyte ratio (MO%) (Table 3). Recently, research results have shown the MDW value of greater than 20.0 U is effective for sepsis detection [25]. Our findings were consistent with them (AUC 0.767).

In the diagnosis of sepsis in patients with cirrhosis (Table 4), the most sensitive of the three traditional biomarkers was sCD163. Among the VCS parameters, the diagnostic index (Youden index) of MMV and MDW was the highest. SCD163 is a marker of macrophage activation, and macrophages originate primarily from peripheral circulating monocytes and are major members of the natural immune system [26]. When macrophages were activated by infection and tumor, the expression of CD163 molecules on macrophage membrane was increased, and the content of sCD163 in serum was increased. Of all the diagnostic parameters, the best predictor of sepsis in patients with cirrhosis was IL-6, with a diagnostic index (Youden index) of 74.5%. This may be related to the timely collection and testing of blood. Of course, the combined diagnosis of MMV and MDW would play a predictive role in the early diagnosis of sepsis in patients with cirrhosis, and its diagnostic indexes were found to be increased to 62.89%.

Furthermore, IL-6 also appeared to be the best predictor of infections in patients with cirrhosis among all diagnostic indicators in the ROC curve (Figure 3). At the cutoff value of 392 pg/mL, the sensitivity was 70.53% and the specificity was 84%. The second was MMS. When the cutoff value was 89.53, the sensitivity and specificity were 77.89% and 72%, respectively. Among the VCS parameters, MNV in combination with MMS could improve diagnostic performance (AUC 0.82) and early rapid detection in patients with infections. And the MNV+MMS diagnostic index was 78.32, which was more favorable with PCT. It was also worth pointing out that its specificity was also relatively higher.

The study still has some limitations to consider. First, a larger study is needed to determine whether VCS parameters make better sense during prolonged monitoring. This includes monitoring early and appropriate antibiotic therapy, the relationship between changes in VCS parameters and the length of hospitalization in ICU patients with sepsis and infections, and their relationship with morbidity and mortality. Second, there is currently no gold standard for the diagnosis of sepsis, and misclassification of sepsis and nonsepsis inevitably limits the accuracy of biomarkers [27]. Although the study was based on the newly established “septic-3” standard, it did not distinguish between severe sepsis and septic shock. Third, the study examined the etiology of sepsis and localized infections in patients with cirrhosis (e.g., bacterial, fungal), but fungal infection accounted for 10.71% and 8.24% in the two groups, respectively. Because of its small sample size, no distinction was made between fungal and bacterial infections to compare the diagnostic efficacy of biomarkers and VCS parameters. In addition, the relatively small groups and high selection (sepsis and limited organ-specific infections) of patients with cirrhosis might have led to incorporation bias that overestimated the diagnostic capacity of the studied markers. Therefore, more prospective cohort studies are needed to further verify the clinical usefulness of VCS parameters in patients with cirrhosis with infections. In group selection, sepsis and systemic inflammatory response syndrome should be compared, and severe sepsis and septic shock should be also distinguished, which may have an effect on VCS parameters.

In conclusion, VCS parameters have the potential to be used to evaluate and predict early infections in patients with cirrhosis. In the diagnosis of sepsis and limited infections in patients with cirrhosis, the combination of VCS parameters can increase the sensitivity and specificity of diagnosis.

## Figures and Tables

**Table 1:** Blood culture data for 28 cases in the sepsis group (52%).

Pathogen species	Number of cases	Percentage of total (%)
*Klebsiella pneumoniae*	7	25.00
*Pseudomonas aeruginosa*	5	17.86
*Acinetobacter baumannii*	4	14.30
*Escherichia coli*	3	10.71
*Pseudomonas cepacia*	3	10.71
*E. serrata*	2	7.14
*Klebsiella-producing acid*	1	3.57
*Candida albicans*	1	3.57
*Candida glabrata*	1	3.57
*Candida krusei*	1	3.57

**Table 2:** Body fluid and secretion culture data for 85 cases in the infection group.

Pathogen species	Number of cases	Percentage of total (%)
*Klebsiella pneumoniae*	9	10.59
*Pseudomonas aeruginosa*	8	9.41
*Escherichia coli*	7	8.23
*Stenotrophomonas maltophilia*	7	8.23
*Acinetobacter baumannii*	7	8.23
*Enterobacter cloacae*	6	7.05
*Proteus mirabilis*	5	5.88
*Staphylococcus aureus*	4	4.71
*Klebsiella-producing acid*	4	4.71
*Candida albicans*	4	4.71
*Candida krusei*	3	3.53
*Pseudomonas cepacia*	3	3.53
*Candida tropicalis*	3	3.53
*Candida glabrata*	3	3.53
*Xylose oxidizing Alcaligenes*	3	3.53
*Enterococcus faecium*	2	2.35
*Enterococcus faecalis*	2	2.35
*Streptococcus viridis*	1	1.18
*Staphylococcus haemolyticus*	1	1.18
*Staphylococcus epidermidis*	1	1.18
*Aspergillus*	1	1.18
*Mucor*	1	1.18

**Table 3:** Baseline characteristics, inflammatory marker, and VCS parameters.

	Group 1	Group 2	Group 3	All groups	*P* value		
	Sepsis (*N* = 54)	Infection (*N* = 95)	Ctr (*N* = 100)		1 vs. 2	1 vs. 3	2 vs. 3
Age (year)	66.6 ± 16.0	65.5 ± 15.8	64.2 ± 16.3	NS	NS	NS	NS
Sex (m/f)	33/11	56/39	70/30	NS	NS	NS	NS
Procalcitonin (ng/mL)	7.75 ± 8.71	3.28 ± 3.61	0.31 ± 0.20	<0.001	<0.05	<0.001	<0.001
IL-6 (pg/mL)	2742.28 ± 1853.46	720.57 ± 379.34	245.92 ± 140.07	<0.001	<0.05	<0.001	<0.001
sCD163 (mg/L)	4.53 ± 5.89	2.77 ± 2.05	0.78 ± 0.50	<0.001	NS	<0.001	<0.001
WBC (×10^9^/mL)	13.81 ± 7.2	10.21 ± 4.6	5.4 ± 6.2	<0.05	NS	<0.001	0.032
Neutrophil % (NE)	76.7 ± 13.9	75.8 ± 14.2	64 ± 8.2	<0.05	NS	<0.001	<0.001
Monocyte % (MO)	8.06 ± 7.34	7.24 ± 4.56	8.04 ± 3.24	NS	NS	NS	NS
VCS parameters							
Neutrophils							
Volume (MNV)	147.04 ± 10.65	146.76 ± 9.99	140.16 ± 6.47	<0.001	NS	<0.001	<0.001
Conductivity (MNC)	147.85 ± 10.42	146.17 ± 8.47	148.99 ± 7.55	0.073	NS	NS	NS
Scatter (MNS)	142.60 ± 7.04	143.35 ± 5.79	146.05 ± 6.25	<0.05	NS	<0.05	<0.05
Monocytes							
Volume (MMV)	179.52 ± 13.53	170.21 ± 10.40	166.10 ± 10.63	<0.001	<0.001	<0.001	<0.05
Conductivity (MMC)	125.26 ± 6.80	125.95 ± 8.86	124.73 ± 7.07	0.55	NS	NS	NS
Scatter (MMS)	92.18 ± 4.78	91.81 ± 4.27	87.22 ± 4.89	<0.001	NS	<0.001	<0.001
Lymphocytes							
Volume (MLV)	87.96 ± 5.01	88.29 ± 5.00	84.97 ± 5.16	<0.001	NS	<0.05	<0.001
Conductivity (MLC)	115.70 ± 6.81	116.55 ± 5.96	114.78 ± 7.48	0.19	NS	NS	NS
Scatter (MLS)	69.11 ± 6.40	68.31 ± 6.05	68.03 ± 5.28	0.54	NS	NS	NS
Volume distribution width							
Neutrophils (NDW)	20.34 ± 3.71	18.99 ± 2.35	18.37 ± 2.26	<0.001	<0.05	<0.001	NS
Monocytes (MDW)	21.82 ± 4.41	19.02 ± 2.69	18.21 ± 1.99	<0.001	<0.001	<0.001	NS

^∗^ *P* value < 0.05 (ANOVA with post hoc analysis) was considered statistically significant. NS: not significant; Ctr: cirrhosis patients with nonsepsis and no infections; IL-6: interleukin-6; sCD163: soluble hemoglobin scavenger receptor.

**Table 4:** Evaluation of traditional inflammatory biomarkers and VCS parameters in indicating sepsis of patients with cirrhosis by ROC (positive = 54, negative = 100).

Parameter or biomarkers	AUC (%)	Sensitivity (%)	Specificity (%)	PPV (%)	NPV (%)	Cutoff value (positive cases)^†^
PCT (ng/mL)	90.1	77.8	93	85.7	88.6	0.51 (42)
IL-6 (pg/mL)	71.8	81.5	93	86.3	90.3	465 (44)
sCD163 (mg/mL)	84.4	83.3	76	65.2	89.4	0.97 (45)
MNV	71.6	57.4	86	68.9	78.9	143.88 (31)
MMV	79.3	75.9	73	60.3	84.9	170.33 (41)
MMS	62.6	61.1	84	67.3	80.0	92.17 (33)
MLV	67.5	90.7	37	43.8	88.1	81.96 (49)
NDW	71.7	88.9	44	46.2	88.0	17.78 (48)
MDW	76.7	75.9	76	63.1	85.4	19.19 (41)
MMV and MDW	88.6	90.7	95	90.7	95.0	

AUC: area under the curve; NPV: negative predictive value; PPV: positive predictive value; PCT: procalcitonin; IL-6: interleukin-6; sCD163: soluble hemoglobin scavenger receptor; ROC: receiver operating characteristic. ^†^The positive cases were calculated by a cutoff value from respective ROC curves.

**Table 5:** Evaluation of traditional inflammatory biomarkers and VCS parameters in indicating infection of patients with cirrhosis by ROC (positive = 95, negative = 100).

Parameter or biomarkers	AUC (%)	Sensitivity (%)	Specificity (%)	PPV (%)	NPV (%)	Cutoff value (positive cases)^†^
PCT (ng/mL)	89.2	86.32	56	65.08	81.15	0.31 (82)
IL-6 (pg/mL)	83.9	70.53	84	80.72	75.00	392 (67)
sCD163 (mg/mL)	87.7	98.47	45	60.71	81.82	0.61 (85)
MNV	73.3	90.53	38	58.11	84.44	137.81 (86)
MMS	76.3	77.89	72	72.55	77.42	89.53 (74)
MLV	69.4	97.89	27	56.02	93.10	80.88 (93)
MNV and MMS	82.6	86.32	92	82.00	87.62	

AUC: area under the curve; NPV: negative predictive value; PPV: positive predictive value; PCT: procalcitonin; IL-6: interleukin-6; sCD163: soluble hemoglobin scavenger receptor; ROC: receiver operating characteristic. ^†^The positive cases were calculated by a cutoff value from respective ROC curves.

## Data Availability

The data used to support the findings of this study are available from the corresponding author upon request.
